# French public care in ophthalmology: a nationwide survey

**DOI:** 10.3389/fmed.2026.1718848

**Published:** 2026-04-14

**Authors:** Adrian Bord, Tristan Bourcier, Anne-Lise Hirsch, Mathilde Gallice, Bastien Boussat, Pierre-Yves Robert, Christophe Chiquet

**Affiliations:** 1 Department of Ophthalmology, Grenoble Alpes University Hospital, Grenoble, France; 2 Department of Ophthalmology, Strasbourg University Hospital, Strasbourg, France; 3 French Council of University Ophthalmologists (COUF), Paris, France; 4 French Council of Hospital Ophthalmologists (COHF), Paris, France; 5 Department of Ophthalmology, Gonesse Hospital, Gonesse, France; 6 HP2 Laboratory, INSERM U1300, Université Grenoble Alpes, Grenoble, France; 7 Department of Clinical Epidemiology, Grenoble-Alpes University Hospital, Grenoble, France; 8 TIMC UMR 5525 CNRS, Computational and Mathematical Biology Team, Grenoble Alpes University, Grenoble, France; 9 Department of Ophthalmology, Limoges University Hospital, Limoges, France

**Keywords:** epidemiology, eye care, healthcare, hospital, ophthalmology, public health

## Abstract

**Aim:**

To describe the medical resources in ophthalmology departments of French public hospitals and to suggest what training needs could be relevant for the future.

**Methods:**

An online survey was emailed to heads of ophthalmology departments in French public hospitals, including both general and university hospitals. The questionnaire included data on the ophthalmological emergency services, the demographics and the subspecialties declared by practitioners. Data were statistically analyzed geographically, across administrative areas, in university and non-university hospitals.

**Results:**

One hundred and forty-five centers replied to the questionnaire, representing 823 ophthalmologists, of whom 408 were men (49.6%). The mean age of ophthalmologists was 42.4 ± 5.3 years, with 57% of the physicians being under 40 years old and 14% over 60 years old. The medical positions were distributed as follows: hospital practitioners (*n* = 335; 40.7%), mixed practitioners (*n* = 179; 21.7%), fellows (*n* = 156; 18.9%), university fellows (*n* = 75; 9.1%), professors (*n* = 63; 7.7%), and associate professors (*n* = 15; 1.8%). The mean density of public ophthalmologists was 1.37 per 100,000 inhabitants (range: 0–8.4). The distribution of ophthalmologists across the country was characterized by a notable representation of subspecialties such as retina, glaucoma, and cataract surgery. However, the distribution of ophthalmologists specializing in neuro-ophthalmology and ocular inflammation exhibited significant variability.

**Conclusion:**

This study highlights significant geographical disparities in the distribution of public ophthalmological care in France, especially in neuro-ophthalmology and ocular inflammation. This preliminary work will facilitate the adjustment of the number of practitioners in public hospitals according to the population’s requirement for eye care.

## Introduction

The number, density, training and distribution of ophthalmologists are critical factors in ensuring access to ophthalmologic care, directly impacting the quality of care provided to patients. In France, a country with 68 million inhabitants facing significant demographic and public health challenges, evaluating the geographical distribution and specialization of ophthalmologists is a key public health issue. In 2023, the DREES (Direction for Research, Studies, Evaluation, and Statistics) recorded 5,749 ophthalmologists, with 3,412 practicing privately, 1,316 in mixed practice and 483 working exclusively in public hospitals.^1^ This corresponded to 8.45 ophthalmologists per 100,000 inhabitants. However, these data did not inform us of the subspecialty and the geographical distribution of the medical resources available in public hospitals.

Some recent studies have reported geographical variations in the availability of ophthalmologic care. For instance, a worldwide study conducted in 2020 (1) highlighted significant disparities in the distribution of ophthalmologists based on gross domestic product. The study reported a range from 3.7 ophthalmologists per million inhabitants in economically disadvantaged countries and 76.2 in economically developed countries. A recent study from the United States (2) examined the geographical distribution of US ophthalmic subspecialists (including the scope of cornea, glaucoma, oculoplastic, retina and strabismus), with a particular focus on rural practice patterns over time.

A number of studies conducted in France (3–5), coordinated by the French Council of University Ophthalmologists (COUF), revealed significant geographical disparities in the provision of care for pediatric, glaucoma, and vitreoretinal surgery. These studies provided data on the epidemiology of specialized surgeons and highlighted the need to increase training capacity to ensure a sufficient number of practitioners in these fields in the long term, considering future retirements. In France, there is a paucity of geographical description of public ophthalmologists, especially with regard to the type of activities and subspecialities. To facilitate proactive recruitment in the public sector, it is imperative to enhance our understanding of data pertaining to ophthalmologists, including their demographic characteristics (age, gender, status) and their role within the healthcare system.

The objective of the present study was to provide a comprehensive description of the epidemiological data, geographical distribution and subspecialties of ophthalmologists in French public hospitals in 2023. The present study was based on a national survey sent to the heads of ophthalmology departments in French public hospitals and university hospitals.

## Materials and methods

A questionnaire consisted of 28 questions divided into three sections (Supplementary Data S1) and covered three main areas: (1) Hospital center identification, (2) information of each hospital center (number of annual emergency and non-emergency consultations, inpatient department availability and number of beds, ophthalmologic emergency services, dedicated operating theaters, ambulatory surgery ratio), and (3) information on post-graduate doctors (10 questions). The study did not include residents. Sub-specialties analyzed in this report included cataract, glaucoma and retina surgery, corneal transplantation, and medical subspecialties (glaucoma, neuro-ophthalmology, ocular surface, ocular inflammation).

The final version of the questionnaire was prepared on an electronic platform [SPHINX software (version 4.27, 2024)] and sent from July 2023 to July 2024 to the directors of a Department of Ophthalmology of each public hospital.

**Figure 1:**
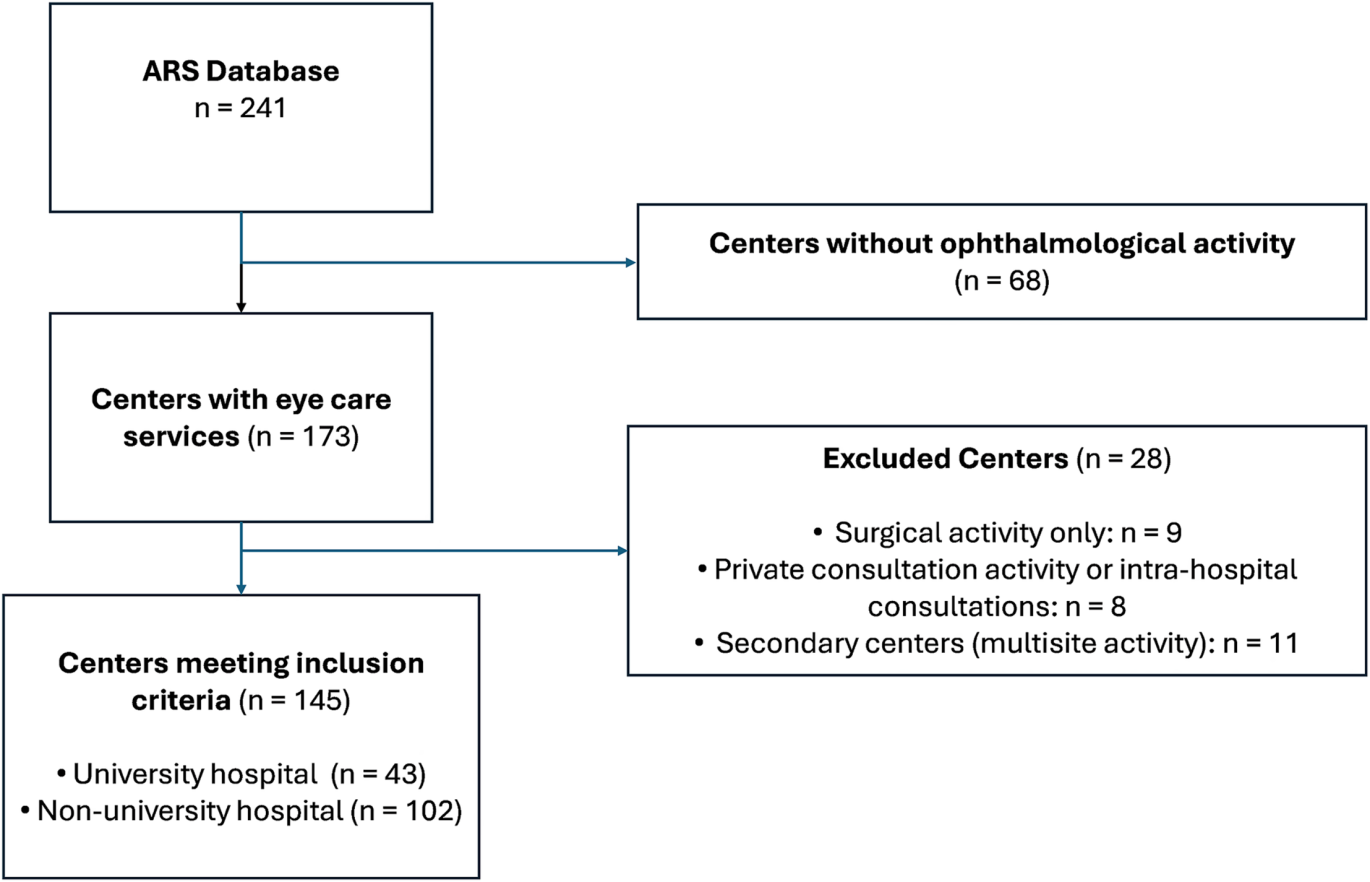
Flow chart of the selection of 145 public hospital centers which provided ophthalmological care in 2023.

The study included centers with at least one outpatient availability. The ARS (Regional Health Agency) listed 241 centers employing at least one ophthalmologist in 2022, including 43 university hospitals. Hospitals with ophthalmological surgical activity and no outpatient availability (*n* = 9), and centers with only private consultations or intra-hospital referrals (*n* = 8) were excluded from the analysis. In Centers sharing activities with a main hospital center (“multisite” center, *n* = 11), the medical staff was only reported in the main hospital center. Figure 1 is a flow chart summarizing the identification of public hospital centers with eye care in France.

Public health establishments in France include university hospital centers and hospital centers. Public hospitals were identified from data published by the ARS in 2022. The study focused on metropolitan France and data were summarized at the level of 96 administrative areas. Data from practitioners were anonymized and consent from the Head of the Ophthalmology department was obtained for the use and publication of the collected data.

### Statistical analysis

The collected data were analyzed using descriptive parameters (mean, standard deviation, median, interquartile IQR 25–75%). The results were then presented graphically, and the geographical distribution of subspecialties was mapped using QGIS software (Version 3.36, 2024). We graphically represented the departments based on the following categories: absence of data (value = 0), first quartile (IQR 1–25%), second and third quartiles (IQR 25–75%) and the last quartile (IQR 75–100%).

## Results

**Figure 2:**
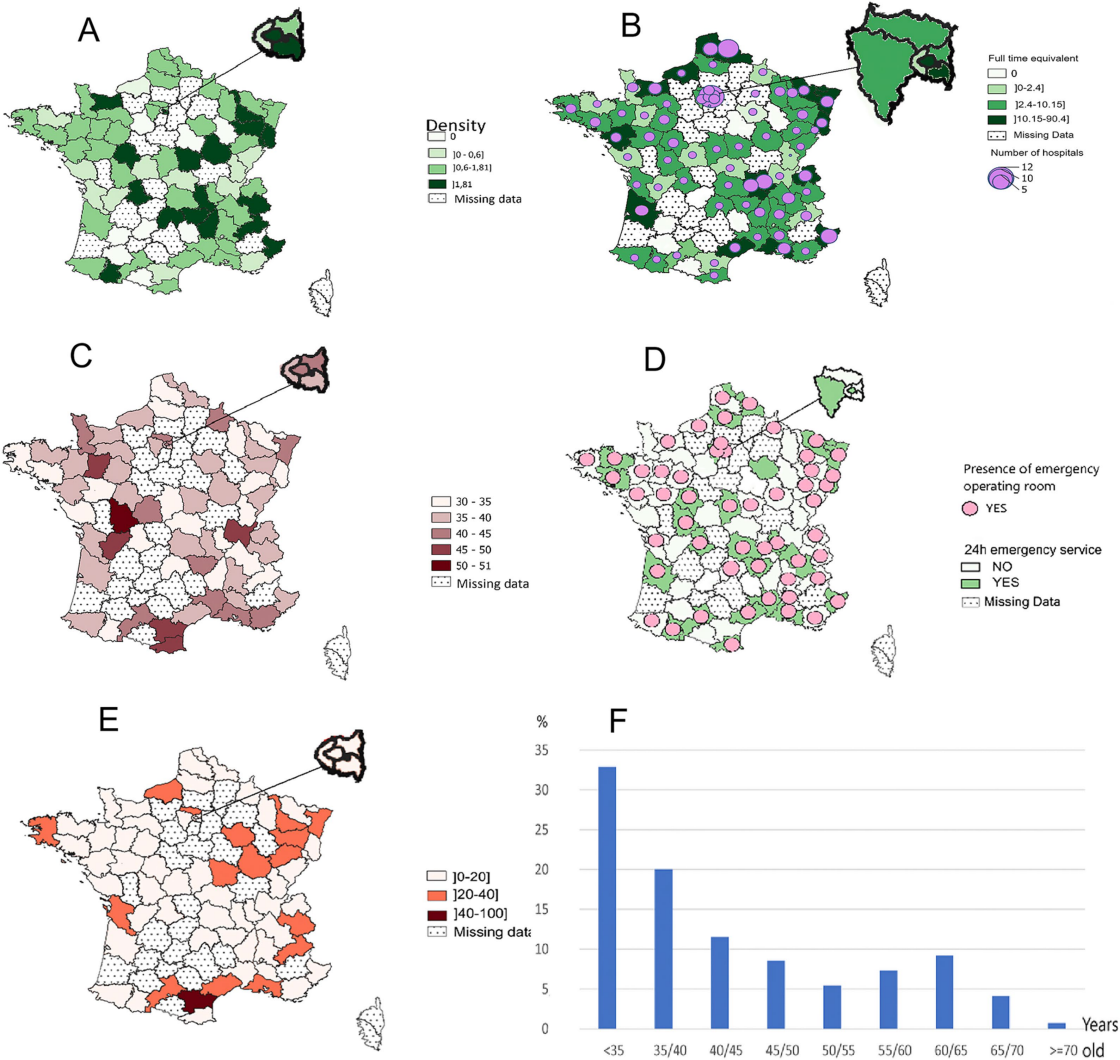
Epidemiological data of 813 ophthalmologists from 145 public hospital centers. **(A)** Density of ophthalmologists per 100,000 habitants. **(B)** Number of public hospitals and full-time equivalents. **(C)** Median age of ophthalmologists. **(D)** 24-h emergency department and emergency operating rooms. **(E)** Percentage of ophthalmologists over 60 years old. **(F)** Age distribution of ophthalmologists in public hospitals.

From 145 centers, we collected data from 859 post-graduate ophthalmologists; with 4% of missing data (*n* = 36). Missing data were noted in 23/96 administrative areas and were related to 29 non-university hospitals. These corresponded mainly to small public hospitals with one or two ophthalmologists and large hospitals in five cities with populations of more than 70,000. Figures 2A,B illustrate the distribution of public hospitals and emergency units in France.

Epidemiological data from the 823 ophthalmologists (Figure 2) showed a sex ratio of 0.98, with 415 women (50.4%) and 408 men (49.6%). The average age was 42.4 ± 5.3 years and median age 38 years (IQR 33–50) The total FTE represented by the 823 ophthalmologists was 590, implying an average individual FTE of 0.72 (590/823), primarily due to part-time work. Mixed practitioners have a mean FTE of 29.6% at the hospital (*n* = 179). Ophthalmologists were mostly located in large cities (Figure 2). The average density of ophthalmologists was 1.37 per 100,000 inhabitants and ranged from 0 in administrative area #48 to 8.47 in administrative area #75 (i.e., Paris, median = 1.17 ± 1. IQR 0.6–1.81).

Overall, half of practitioners were under 40 years old, representing 53.3% of the medical staff (Figure 2). Fellows in public hospital and university hospital (*n* = 231) accounted for 57% of this group. Practitioners over 60 account for 14% of the total medical staff. The national distribution of ophthalmologists showed disparities in median age according to the department, with a median age increasing in the southern regions. The medical positions were distributed as follows: hospital practitioners (*n* = 335; 40.7%), mixed practitioners (*n* = 179; 21.7%), fellows (*n* = 156; 18.9%), university fellows (*n* = 75; 9.1%), professors (*n* = 63; 7.7%), and associate professors (*n* = 15; 1.8%).

**Figure 3:**
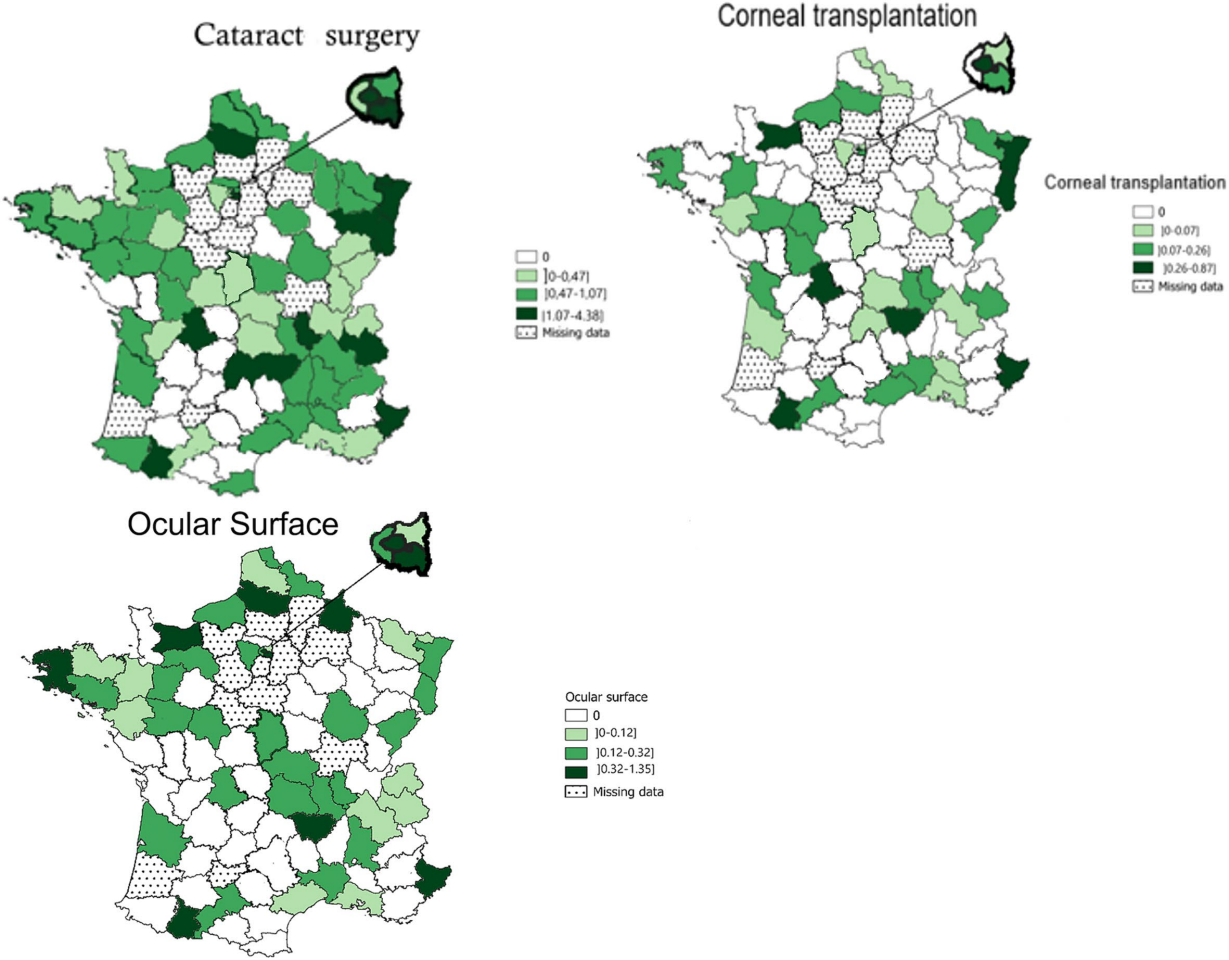
Geographical distribution of ophthalmologists in public hospitals for the anterior segment: cataract surgery; corneal transplantation; and ocular surface. Bars indicate 25–75% confidence intervals.

The subspecialties of the anterior segment (Figure 3) included, in order of frequency, cataract surgery (the national total of FTEs was 353.4 – IQR: 0.47–1.07 FTE/100,000 inhabitants), ocular surface (86.4 FTEs – IQR: 0.12–0.32/100,000 inhabitants), and corneal transplantation (56.1 FTEs – IQR: 0.07–0.26/100,000 inhabitants). Ophthalmologists with an activity in cataract surgery, ocular surface, corneal transplant, contact lens practice, and refractive surgery were absent in 8, 31, 38, 39, and 47 administrative areas, respectively. Fifty nine percent of all public ophthalmologists performed cataract surgery, most of them being in general hospitals. Ten percent of all public ophthalmologists performed corneal transplantation, mainly in university hospitals (Table 1).

**Table 1:** Density of ophthalmologists in university (urban areas) and general hospitals (rural areas) in France.

Full time equivalents	Public hospitals	University hospitals		General hospitals	
		Total	Per 100,000 habitants	Total	Per 100,000 habitants
FTE	353.4	136.6 (38.7%)	0.209	216.8 (61.3%)	0.331
Cataract surgery (FTE)	353.4	136.6 (38.7%)	0.209	216.8 (61.3%)	0.331
Corneal graft (FTE)	56.1	35 (62.4%)	0.053	21.1 (37.6%)	0.032
Ocular surface (FTE)	86.4	46.6 (41.5%)	0.071	39.8 (46.1%)	0.061
Surgical glaucoma (FTE)	101.75	41.5 (40.8%)	0.063	60.2 (59.2%)	0.092
Medical glaucoma (FTE)	182.7	49.3 (27%)	0.075	133.4 (73%)	0.204
Neuro-ophthalmology (FTE)	55.6	25.8 (46.4%)	0.039	29.8 (53.6%)	0.045
Medical retina (FTE)	238.6	87.4 (36.6%)	0.133	151.2 (63.4%)	0.231
Surgical retina (FTE)	147.7	71.2 (48.2%)	0.109	76.5 (51.8%)	0.117
Inflammation (FTE)	238.6	87.4 (36.6%)	0.133	151.2 (63.4%)	0.231

FTE, full-time equivalent.

**Figure 4:**
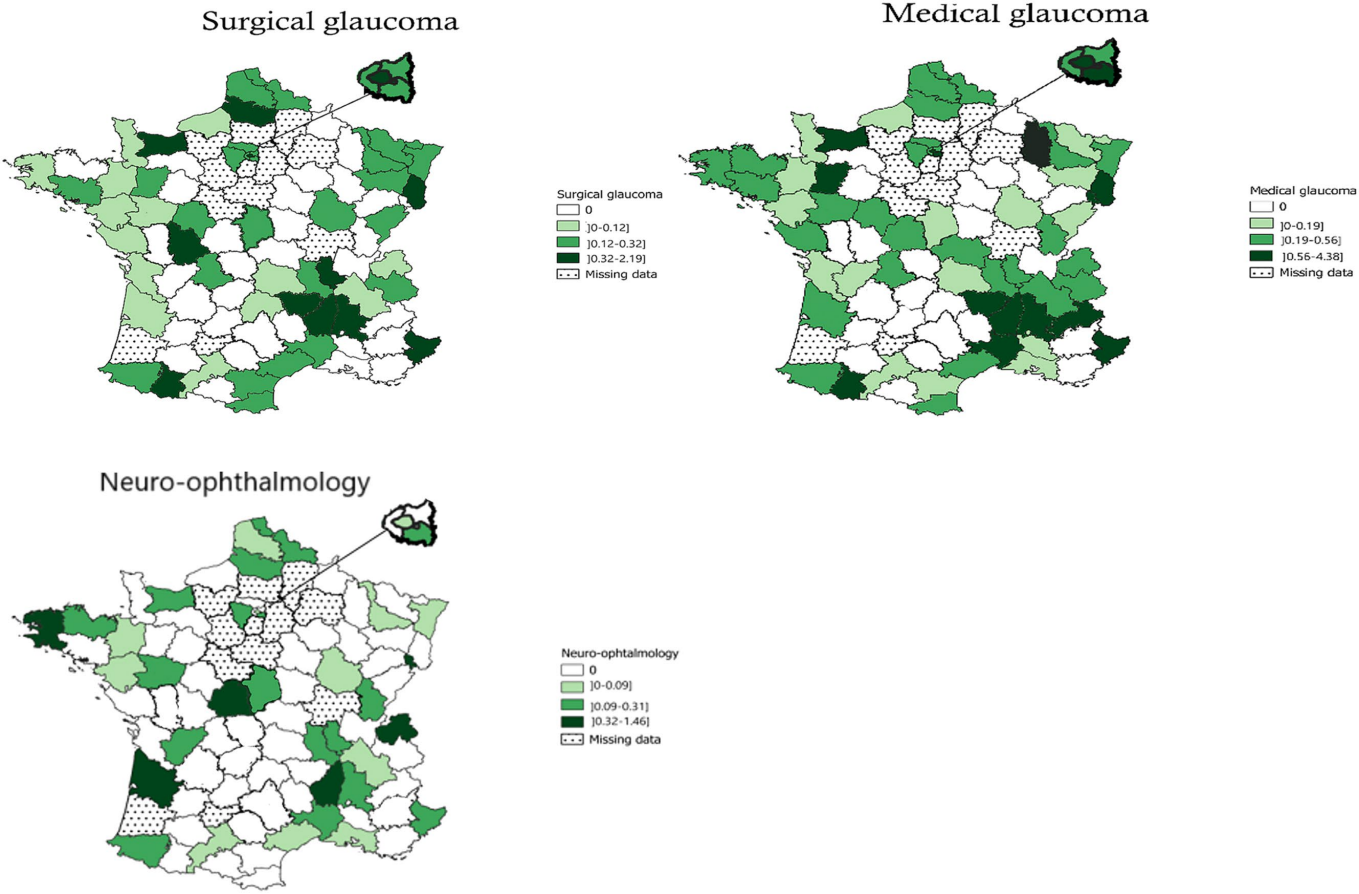
Geographical distribution of ophthalmologists in public hospitals for surgical glaucoma, medical glaucoma, and neuro-ophthalmology. Bars indicate 25–75% confidence intervals.

The subspecialties of optic nerve diseases (Figure 4) included, in ascending order, medical glaucoma (182.7 FTEs – IQR: 0.19–0.56 FTE/100,000 inhabitants), surgical glaucoma (101.7 FTEs – IQR: 0.12–0.32/100,000 inhabitants), and neuro-ophthalmology (55.6 FTEs – IQR: 0.09–0.31/100,000 inhabitants). Ophthalmologists with an activity in medical glaucoma, surgical glaucoma, and neuro-ophthalmology were absent in 17, 25, and 43 administrative areas, respectively. In medical glaucoma, 27% of ophthalmologists worked in university hospitals, 73% in non-university hospitals, 18.7% of all public ophthalmologists performed surgical glaucoma.

**Figure 5:**
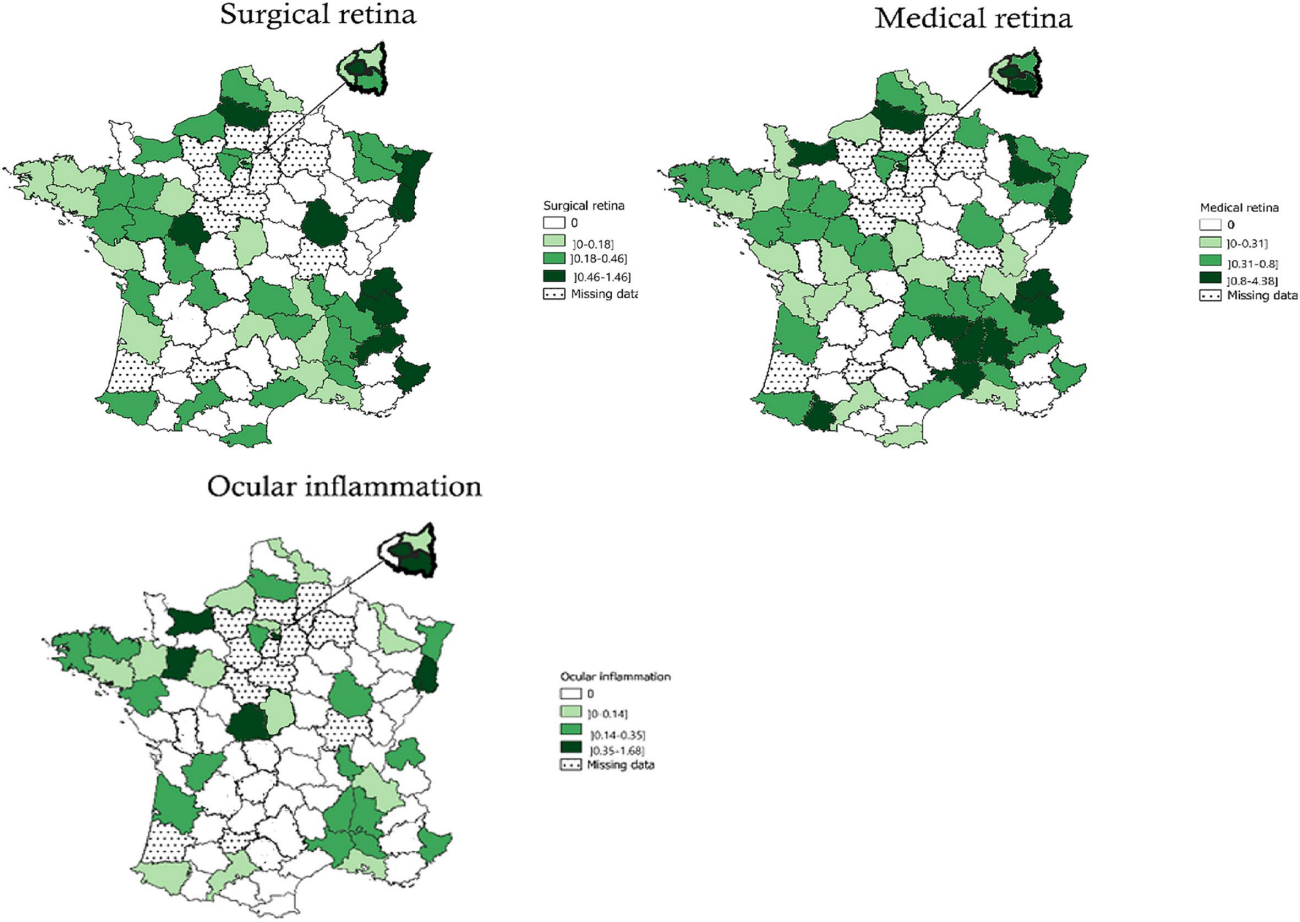
Geographical distribution of ophthalmologists in public hospitals for surgical retina, medical retina, and ocular inflammation. Bars indicate 25–75% confidence intervals.

The subspecialties of the retina (Figure 5) included medical retina (the national total of FTEs was 182.7 FTEs – IQR: 0.31–0.80 FTE/100,000 inhabitants), surgical retina (101.7 FTEs – IQR: 0.18–0.46/100,000 inhabitants), and ocular inflammation (238.6 FTEs – IQR: 0.14–0.35/100,000 inhabitants). Ophthalmologists with an activity in medical retina, surgical retina, and ocular inflammation were absent in 14, 24, and 38 administrative areas, respectively. 41.2 and 24.3% of all public ophthalmologists performed medical, or surgical, respectively. 14.0% of all public ophthalmologists had a clinical activity in ocular inflammation.

## Discussion

The objective of this study was to provide a comprehensive description of the epidemiological data pertaining to ophthalmological public care services in French public hospitals in 2023. The findings of this study highlight significant geographical disparities in the distribution of hospital-based ophthalmologists, particularly in the field of neuro-ophthalmology and ocular inflammation. A major strength of this study lies in the comprehensive nature of the collected data, covering not only some epidemiological data from ophthalmologists but also detailed information on their professional activities (FTE positions and subspecialties). A comprehensive dataset was obtained from 823 ophthalmologists, encompassing both salaried practitioners (*n* = 464) and those engaged in a combination of salaried and private practice (*n* = 359). In comparison, the DREES (see Footnote 1) reported 493 salaried practitioners and 1,316 mixed practitioners. There are some significant discrepancies with our questionnaire-based evaluation, reporting 335 hospital-based practitioners and 179 mixed practitioners. In the present study, we excluded hospital centers with ocular surgery or ocular laser only (always performed by mixed practitioners) and centers offering only consultation for hospitalized patients. The design of the study may have resulted in an underestimation of the number of public hospitals, particularly smaller hospital centers that do not have full-time practitioners. This potential bias may, at least in part, account for the observed differences between this study and the previous reports of the DREES. Finally, this bias is probably low or even negligible, as the ophthalmological medical activities carried out in a hospital for its inpatient services do not cover the needs of the general population and are considered minor. With regard to surgical activities performed in the hospital by surgeons who do not perform any other medical activities in the hospital, this indicates that the surgeon only uses the technical facilities, without participating in the hospital’s medical activities or offering consultation services. Thus, these two types of activity are considered marginal and not counting them does not result in significant bias in the assessment of general ophthalmological resources.

### Age of practitioners in public hospitals

The median age of hospital-based practitioners in our study was 38 years old (IQR 33–50) and was related to the proportion of young practitioners including university and non-university fellows (*n* = 251; 30.4%). Most fellows will practice in private centers after 1 or 2 years of fellowship. In contrast, hospital-based practitioners, who are public service employes, had a median age of 40 years old (IQR 35–54). This age distribution highlights the necessity to anticipate training of ophthalmologists to replace the 14.6% of practitioners over 60 years of age who are likely to retire in the next 5 years. This issue is also observed in Canada, where there are concerns about the capacity to train enough professionals to meet the increasing demand driven by the aging baby boomer population, despite maximum training capacity. The concern is not so much about the ophthalmologist-to-population ratio declining overall, but rather the ratio of ophthalmologists to people over 65 (6).

### Sex ratio of practitioners in public hospitals

The profession is experiencing a gradual feminization, in all positions of the public health service. For hospital-based practitioners, the frequency of women was previously reported 48% in 2021, 44% in 2023 (see Footnote 1) and 54% in the present study. This trend was also noted for university positions, since the rate of women was 44% in 2021 and 47% in 2023 for Associate Professor-Hospital practitioner, and 21% in 2021 and 33% for Full Professor-Hospital practitioner, respectively. The feminization of ophthalmologist in private practice has also been documented with 52% of new private practice ophthalmologists being female. An American study explored gender differences in subspecialty choices, revealing an overall male predominance in 2020 (66%). However, women made up a higher proportion in specific fields, such as pediatrics (72%) and glaucoma (59%). Conversely, only 32% of vitreoretinal surgeons were women. A recent epidemiological American study (2) confirmed globally a predominance of male (72.6%) for cornea, glaucoma, oculoplastic and retina. In France, data in 2016 (5) also showed a lower prevalence of women in vitreoretinal surgery (39%). Our results in 2023 indicate similar trend, with a lower prevalence in vitreoretinal surgery (30%) and corneal surgery (30%), while the representation of women in glaucoma is nearly equal (48%). These data showed that there is a trend toward feminization in ophthalmology, which varies according to subspecialty and country.

### Number of practitioners and density in metropolitan France

The national average of hospital-based ophthalmologist density is 1.04 per 100,000 inhabitants but this varies significantly by administrative area, from absence of practitioners in some areas to 8.5 practitioners/100,000 inhabitants in Paris. Our results are consistent with the DREES 2021 report (see Footnote 1), which reported in 2023 a total density of 8.47, with 0.73 for hospital-based practitioners, 1.94 for mixed practitioners, 5.11 for private practitioners, and 0.64 for other salaried practitioners. Despite some missing data, our results suggest that the distribution of hospital-based ophthalmologists was more balanced in 2023 as compared to the findings in the DREES 2021 report. Some areas, particularly those along the “diagonal of emptiness” (such administrative areas #52, 48, 09, 89), and those in the Southwest (#32, 46, 47) remain under-resourced. Some improvements are documented, such as the areas # 01 and 85, which lacked hospital-based ophthalmologists in 2021, and have at this time ophthalmologists practicing in these areas (*n* = 4). An increase in the number public practitioners was also noted in administrative areas such as #55 (from 1 to 3), #65 (from 2 to 5), #15 (from 1 to 6), and #07 (from 2 to 7).

The *regional disparities* observed in France are also described in other countries. For instance, a 2020 Chinese study showed that 85% of ophthalmology hospitals were located in urban areas (7). This study, which involved 31 provinces and 395 specialized hospitals (16% in the public sector), found that both private and public activities were concentrated in eastern (53%) and central (33%) China. Similarly, in Ontario, Canada, in 2014 (8), the ophthalmologist-to-population ratio was 3.35 per 100,000 inhabitants, but this varies significantly by region, from 0.89 to 5.4. In the United States, a 2022 study (U. S. Physician Workforce Data Dashboard. AAMC^2^) reported a national density of 5.4 per 100,000 inhabitants, ranging from 2.2 to 13.8. Comparisons between France and large countries is limited by the fact that definition of rurality is somewhat very different in large countries such as China, US, Australia as compared to European countries, such as France. For instance, it is generally estimated that one of the most remote villages is located approximately 50 to 75 miles from a large city (i.e., with a population of over 200,000). France is densely populated and well connected (roads, medium-sized cities, services). This is therefore very different from United States and Australia (300–600 miles, 8–12 h of driving). Another important difference between France and Anglo-Saxon countries is that there are no optometrists in France and that access to healthcare, particularly for optical correction, is mainly provided by ophthalmologists. In Canada, the lower density of ophthalmologists can be partially offset by the high optometrist workforce (≈16.5 per 100,000), reflecting a shared-care model that could buffer access despite fewer ophthalmologists (9). Finally, it should also be borne in mind that this study concerns healthcare provision in public hospitals and that the private sector has not been taken into account. The private sector is particularly important in France and can compensate for the lack of healthcare provision in the public system in certain regions.

*Regarding multisite practice*, our study reveals that approximately 25% of practitioners share their ophthalmological activity between two centers and maintain a medical presence in under-resourced areas.

*Medical retina* activity was well represented nationwide, with a total of 246 full-time equivalent representing 41.2% of ophthalmologists practicing this specialty. In a 2016 study using the SNDS database, we identified 202 vitreoretinal surgeons in public hospital centers, which is consistent with 195 surgeons reported in this report. In 2016, the absence of vitreoretinal surgery concerned 9/96 administrative areas (9%) (5). In the present study, approximately 67% of the administrative areas benefitted from public centers for vitreo-retinal surgery.

*Incidence of cataract surgeries* has increased from 826,000 in 2016 to 1,076,216 in 2023. The hospital-based surgical capacity appears adequate, with approximately 400 full-time equivalents evenly distributed across the country. However, cataract surgery was not performed in 8 administrative areas. *Corneal transplantation* was done by 58.7 full-time equivalents, which are well distributed across the country. In a 2016 SNDS study (submitted publication), based on the 5,172 surgical procedures performed in public or private surgeons, the rate of surgery performed in the public sector was 73.5%. Data from the present study are similar to that found in 2016, especially for the number of administrative areas without corneal transplantation (*n* = 35), the number of public surgeons (*n* = 82), their age (39 ± 11.06 years) and the sex ratio (women in 34% out of the cases).

*For glaucoma* surgery, the 2016 study (3) indicated that most surgeons (private or hospital based) were over 55 years old in 18 administrative areas. Today, we observe that in these 18 administrative areas, five have no public glaucoma surgeons.

We acknowledge many limitations of our study, including the declarative nature and missing data. The potential bias associated with 4% missing data for general hospitals may be considered as small. Secondly, the activity of each practitioner was evaluated using the full-time equivalent, including general ophthalmology and most of the time one or several subspecialties. We could not quantify the time spared by each practitioner for each subspecialty. This approach might overestimate the availability of subspecialties.

In conclusion, this study provides a detailed overview of ophthalmological care in French public hospitals in 2023. The findings reveal significant disparities in resource distribution. Retinal disease, medical glaucoma, and cataract care are subspecialities which are better covered at the national level. Neuro-ophthalmology and ocular inflammation are mainly performed in major cities. This preliminary work will help to adjust the number of practitioners in public hospitals according to the need in eyecare of the population. Defining the need of eyecare is a complex task, and its calculation should consider epidemiological data of the French population, especially data of the main ocular diseases and their medical and/or surgical management.

## Data Availability

The raw data supporting the conclusions of this article will be made available by the authors, without undue reservation.

## Footnotes

1 https://drees.shinyapps.io/demographie-ps/ (Accessed May 30, 2024).

2 https://www.aamc.org/data-reports/report/us-physician-workforce-data-dashboard (Accessed September 2, 2024).

## Group members of French Council of University Ophthalmologists (COUF)

Angioi Karine, Arndt Carl, Arnould Louis, Audo Isabelle, Baillif Stéphanie, Baudouin Christophe, Behar-cohen Francine, Beral Cindy, Bonnet Clemence, Bodaghi Bahram, Borderie Vincent, Bouheraoua Nacim, Bourcier Tristan, Bourges Jean-louis, Bremond Dominique, Brezin Antoine, Burillon Carole, Cassoux Nathalie, Chiambaretta Frédéric, Chiquet Christophe, Cochener Béatrice, Conart Jean-baptiste, Couturier Aude, Creuzot garcher Catherine, Daien Vincent, Daruich Alejandra, David Thierry, Delyfer Marie-noëlle, Denis Philippe, Denoyer Alexandre, Dormegny Lea, Dot Corinne, Ducloyer Jean-baptiste, El chehab Hussam, Fenolland Jean -rémi, Fournie Pierre, Froussart-maille Françoise, Gabison Eric, Gabrielle Pierre-henri, Gain Philippe, Garcin Thibaud, Gaucher David, Giraud Jean-marie, Giocanti Audrey, Gauthier Anne-sophie, Gueudry Julie, Kanav khanna Raoul, Kodjikian Laurent, Korobelnik Jean-françois, Labalette Pierre, Labbe Antoine, Labetoulle Marc, Lebranchu Pierre, Lemeur Guylène, Leveziel Nicolas, Matet Alexandre, Mathis Thibaud, Martel Arnaud, Merle Harold, Meunier Isabelle, Miere Alexandra, Monnet Dominique, Mouriaux Frédéric, Muraine Marc, Nordmann Jean-philippe, Paques Michel, Pisella Pierre-jean, Quintyn JEAN claude, Robert Pierre-yves, Robert Mathieu, Rousseau Antoine, Sauer Arnaud, Schweitzer Cedric, Vasili Smirnov, Soler Vincent, Souied Eric, Thuret Gilles, Touboul David, Touhami Sarah, Touitou Valérie, Tran Chau, Villain Max.

## Group members of French Council of Hospital Ophthalmologists (COHF)

Abada Samir, Aboudib Antoine, Abry Florence, Adrar ryad, Augey Frederic, Aury Patrick, Benjelloun Fatiha, Berramdane Lotfi, Bihl Lionel, Bollot goutorbe Martine, Boulicot seguin Celine, Brehon Alexia, Bui Quoc, Bussat Aurore, Caputo Georges, Chaabane Molka, Chahed Sadri, Chalalen Mohamed, Charef Bouchra, Clement Manon, Coupier Laurent, Milea Dan, Degeling Hannah, Demailly François-xavier, Donnio Angelique, Duris mehou loko Aina, Faraj Hani, Galatoire Olivier, Gauthier Anne sophie, Gobert Frederic, Gohier Philippe, Grenard Nicolas, Grenot Marine, Guillaumie Trémeur, Haslé Denis-paul, Hirsch Annelise, Houmad gillet Naila, Ichalalen Mohamed, Jany Benjamin, Jaulerry Stephane, Jeanjean Luc, Khalil Antoine, Koch Clémence, Konqui Philippe, Labbé Antoine, Lachkar Yves, Lambrecq Stéphanie, Lamiot Anne katherine, Larré Isabelle, Law-koune Sandrine, Le meur Guylène, Ligeon ligeonnet Patrick, Louza Sarah, Maillard Patrick, Marsal Caroline, Meyer Franck, Moritz Francois, Nasica Xavier, Nenciu Adrian, Nguyen Anh ngoc, Ostendorf Marlene, Payan Jacques, Perone Jean marc, Perrot Chretien, Petry Laurent, Pipelart Valentin, Pollet-villard Frédéric, Puech Cindy, Rabot Alexandra, Remond Perrine, Rescourio Marjorie, Ricouard Fanny, Rochet Elsa, Saihi Emna, Savy Olivier, Slim Malek, Spire Myriam, Tazouta Mohamed karim, Terzic Zoran, Tevidic Patrick, Tonini Matthieu, Tran Anthony, Vignal clermont Catherine, Williamson Wilfrid, Zini Pascale.

## Supplementary material

Supplementary Data S1 Questionnaire consisted of 28 questions divided into three sections (1) Hospital center identification (FINESS number), (2) information of each hospital center (number of annual emergency and non-emergency consultations, inpatient department availability and number of beds, ophthalmologic emergency services, dedicated operating theaters, ambulatory surgery ratio), and (3) information on post-graduate doctors (age, gender, specialties, full time equivalent (FTE), activities across centers, private practice, medical expertise, participation in humanitarian missions).
