# Supplementary material for: French public care in ophthalmology: a nationwide survey
Source: Front Med (Lausanne). 2026 Apr 14;13:1718848. doi: 10.3389/fmed.2026.1718848 (PMC13123254; doi:10.3389/fmed.2026.1718848)
Supplement: Supplementary Data S1 — Questionnaire consisted of 28 questions divided into three sections (1) Hospital center identification (FINESS number), (2) information of each hospital center (number of annual emergency and non-emergency consultations, inpatient department availability and number of beds, ophthalmologic emergency services, dedicated operating theaters, ambulatory surgery ratio), and (3) information on post-graduate doctors (age, gender, specialties, full time equivalent (FTE), activities across centers, private practice, medical expertise, participation in humanitarian missions). [file Supplementary_Data_S1.DOCX]

**OPHTAFORM Questionnaire**

Dear colleague

You are invited to participate in a questionnaire concerning medical doctors practising in the public hospital service. The French Council of University Ophthalmologists (COUF) and the French Council of Hospital Ophthalmologists (COHF) are interested in medical demographics. In 2016, we asked you for information on the number of doctors and some demographic data in order to better understand the training needs of young ophthalmologists and anticipate training needs across the country. This led to several international publications on surgical activity in glaucoma (doi: 10.1111/aos.14916), retina (doi: 10.1111/aos.15143), paediatrics (doi: 10.1016/j.jeph.2024.202786) and cornea (publication submitted).

We would like to send a questionnaire to the heads of departments in hospitals and university hospitals. This questionnaire is quick and easy to complete and will provide a snapshot of human resources in public services in all regions of France. The data is completely anonymous and corresponds to a few demographic details, the type of subspeciality practised and the status of activity.

This data will be analysed with the help of statisticians (Dr Bastien Boussat, MCU-PH, Magali Bouisse, Université Grenoble-Alpes) and will be the subject of a medical thesis (Adrian Bord, Université Grenoble-Alpes).

The data collected will be published in a scientific journal and, if you agree, we will credit you as a participant in the study group.

Before completing the online questionnaire, please find attached a PDF template containing the main data to be provided, with a standard template for each doctor. It may be useful to have your colleagues fill in this information before completing the online questionnaire.

We thank you in advance and remain at your disposal should you have any questions.

Yours sincerely,

Prof. Christophe CHIQUET and Prof. Tristan BOURCIER for COUF Dr AnnelLise HIRSCH for COHF

cchiquet@chu-grenoble.fr, tristan.bourcier@chru-strasbourg.fr, [annelise.hirsch@ch-gonesse.fr](mailto:annelise.hirsch@ch-gonesse.fr)

**I The hospital**

1 Your email address

2 What is the name of your institution

3 In which city is your institution located

4 Its postcode

5 FINESS number

6 Name of the head of department

7 Do you give your permission to be included in the list of participants that will be associated with the publication of the work?

**II Ophthalmology department (2023 data)**

**1 How many senior ophthalmologists with doctorates practise in your department** (Professor, Assistant professor, hospital practitioner, Fellow)

**2 Do you have a hospital ward dedicated to ophthalmology?**

**3 How many beds does it have?**

**4 Do you have an ophthalmology emergency department?**

- Open 24 hours a day
- Open during working hours with an on-call line outside these hours
- Emergency department open only during business hours
- No

**5 How many emergency consultations per year in 2023?**

**6 How many non-emergency consultations per year in 2023?**

**7 Do you have access to operating theatres (scheduled or emergency procedures)?**

**8 What is the volume of outpatient surgery?**

**9 Number of half-days the operating theatre is open per week (e.g. two operating theatres open all day = 4 half-days):**

**10 Number of operating theatres dedicated in part to ophthalmology (excluding emergency theatres)?**

**11 Do you have access to operating theatres in emergencies (at night, at weekends)?**

**III Doctor**

The following sections deal exclusively with doctors who have at least some clinical activity within the facility. Doctors who only use the technical facilities are excluded. **This section si completed for each medical doctor in your department.**

**1 Age**

**2 Gender**

**3 Sub-speciality**

- No
- Lens surgery
- Ocular surface
- Contact lens practice
- Corneal transplant
- Refractive surgery
- Medical glaucoma
- Surgical glaucoma
- Neuro-ophthalmology
- Ocular electrophysiology
- Paediatric ophthalmology
- Strabology for children and adults
- Ocular inflammation
- Ocular oncology
- Orbital surgery
- Eyelid and tear duct surgery
- Low vision
- Ophthalmogenetics
- Expert B-mode ultrasound
- Medical Retina
- Surgical retina
- Other:

**4 Percentage of full-time work: %**

**5 Administrative Status:** (Professor, Assistant professor, hospital practitioner, Fellow)

**6 Activity in another hospital?**

Please provide the names of these hospitals and the percentage of time worked at each centre.

**7 Private practice**

**9 Humanitarian medicine activity**

**10 Do you carry out medical assessments**
